# The family talk intervention for families when a parent is cared for in palliative care – potential effects from minor children’s perspectives

**DOI:** 10.1186/s12904-020-00551-y

**Published:** 2020-04-16

**Authors:** Rakel Eklund, Anette Alvariza, Ulrika Kreicbergs, Li Jalmsell, Malin Lövgren

**Affiliations:** 1 Department of Health Care Sciences, Palliative Research Centre, Ersta Sköndal Bräcke University College, Box 11189, 100 61 Stockholm, Sweden; 2 Capio Palliative Care, Dalen Hospital, 121 87 Stockholm, Sweden; 3 The Department of Women’s and Children’s Health, Paediatric Oncology and Haematology, Karolinska Institutet, Karolinska University Hospital, Astrid Lindgren Children’s Hospital, Childhood Cancer Research Unit, 177 77 Stockholm, Sweden; 4 Department of Breast Cancer, Endocrine Tumours and Sarcoma, Karolinska University Hospital, 171 76 Stockholm, Sweden

**Keywords:** Children as relatives, Children’s self-reports, Minor children, The family talk intervention, Palliative care

## Abstract

**Background:**

Children show long-term psychological distress if family communication and illness-related information are poor during and after a parent’s illness and death. Few psychosocial interventions for families with minor children living with a parent who has a life-threatening illness have been evaluated rigorously. Even fewer interventions have been family-centered. One exception is the Family Talk Intervention (FTI), which has shown promising results regarding increased illness-related knowledge and improved family communication. However, FTI has not yet been evaluated in palliative care. This study therefore aimed to explore the potential effects of FTI from the perspectives of minor children whose parent is cared for in specialized palliative home care.

**Methods:**

This pilot intervention study involves questionnaire and interview data collected from children after participation in FTI. Families were recruited from two specialized palliative home care units. To be included, families must include one parent with life-threatening illness, at least one child aged 6–19 years, and understand and speak Swedish. Twenty families with a total of 34 children participated in FTI; 23 children answered the questionnaire, and 22 were interviewed after participation.

**Results:**

The children reported that FTI increased their knowledge about their parents’ illness. They said the interventionist helped them to handle school-related problems, establish professional counselling, and find strength to maintain everyday life. Children aged 8–12 reported that talking with their parents became easier after FTI, whereas communication was unchanged for teenagers and between siblings. Children also reported having been helped to prepare for the future, and that they benefitted from advice about how to maintain everyday life and minimize conflicts within the family.

**Conclusions:**

Children who participated in FTI reported that it was helpful in many ways, providing illness-related information and improving family communication when a parent has a life-threatening illness. Other potential positive effects reported by the children were that FTI facilitated their preparation for the future, decreased family conflicts, and started to build up resilience.

**Trial registration:**

ClinicalTrials.gov, Identifier NCT03119545, retrospectively registered 18 April 2017

## Background

Children living with a parent who has a life-threatening illness express having difficulty communicating with their parent about their own thoughts and feelings [1]. Children are also known to show psychological distress, years after loss, if communication between the family and health care professionals and illness-related information is poor during the illness and after death [2, 3]. Many parents express a need for support from health care professionals concerning when and how to talk to their children about the illness, but such support is often absent [4–7].

Illness-related information and family communication can be improved by involving the entire family in an intervention [8], but few approaches are based on family systems theory despite the need for them in palliative care contexts [9]. Reviews [5, 10, 11] show that there are few evaluated psychosocial interventions for families with minor children living with a parent with a life-threatening illness. Even fewer of these take a family-centered approach, and many available interventions target children and parents separately, in groups assembled from several families. This, even though family-centered interventions have been suggested to be effective in supporting all family members [8] by improving self-understanding, facilitating the development of new perspectives, and fostering a shared understanding of the illness situation [12–15].

One family-centered intervention that has shown good results for both parents and children is the Family Talk Intervention (FTI) [15–19]. The main goals of FTI are to increase illness-related knowledge and communication within the family, support parenting and improve coping. FTI consists of six key concepts: *Sharing a history together* (to create a common understanding of the illness that includes every family member’s voice and memories); *Bringing knowledge about illness* (to learn about biological causes, diagnosis and treatment); *Addressing the needs of the children* (to draw the children into the process, asking about strengths, skills, interests and relations, and about their knowledge and understanding of the illness); *Planning how to talk to the children* (the parents agree on what and what not to tell the children, and to elicit their questions, worries e.g.); *Breaking the silence together as a family* (to reduce feelings of guilt, fear and shame, and increase knowledge by focusing on the strengths within the family); and *Continuing the family dialogue, moving on and facing the future* (by beginning to understanding the world of others, the family gains a new closeness; this is not easy and requires repeated family meetings and communication) [20]. These concepts are based on different theoretical approaches [13, 14, 20]; psychoeducation increases knowledge about the illness, and through a narrative approach the family members share their stories with each other and create a joint family history. A dialogical approach allows the family to hear each member’s unique perspective and helps maintain a focus on the children’s needs and experiences.

Although most studies have evaluated the effects of FTI in the context of psychiatric care [15–19], the use of FTI has also proven feasible in palliative care, from both children’s and parents’ perspectives (Eklund R, Jalmsell L, Kreicbergs U, Alvariza A and Lövgren M: Children’s experiences of the family talk intervention when a parent is cared for in palliative home care – a feasibility study. under review; Jalmsell L, Eklund R, Kreicbergs U, Lövgren M and Alvariza A: The family talk intervention in palliative home care when a parent with minor children is suffering from life-threatening illness - parents’ perspectives. under review). Children reported that they appreciated the structure and content of FTI and that they felt seen, heard and acknowledged by the interventionists, which established trust and safety (Eklund R, Jalmsell L, Kreicbergs U, Alvariza A and Lövgren M: Children’s experiences of the family talk intervention when a parent is cared for in palliative home care – a feasibility study. under review). Niemelä et al. [21] found that parents with a somatic illness had decreased psychological symptoms after participating in FTI. One study, performed in the context of somatic care, however not in palliative care, have shown that FTI had several benefits for the children, e.g. improved knowledge about the illness, and a perception that the family could talk more openly after participation [22].

FTI has shown promising results in both psychiatric and somatic care: increased illness-related knowledge and improved communication within the family, which are two important areas in palliative care. However, no study using FTI in palliative care has evaluated the potential effects of its use in families with minor children, despite the urgent need for this type of family-centered intervention. Therefore, the aim of this pilot study was to explore the potential effects of FTI, from the perspectives of minor children, when a parent is cared for in palliative care.

## Methods

### Design

This is a pilot intervention study [23] with a post design using data from questionnaires and interviews performed with children who had participated in FTI. The study adheres to CONSORT guidelines.

### The family talk intervention

FTI is manual-based and consists of 6 meetings with some or all family members, led by trained leaders [12, 20], here called interventionists. In this study, the interventionists were a deacon and a social worker, each educated in FTI and with previous clinical experience working with FTI in palliative care. The first two FTI meetings involve only the parents and focus on their history from the time of their diagnosis to the present. The meetings also probe parents’ views on the children’s strengths and needs, and how their situation might have affected the children. The third meeting is between the interventionists and each child, focusing on the child’s experiences of the situation, knowledge about their parent’s illness, feelings, facilitating factors and worries, and any questions the child might have. The fourth meeting includes only the parents and involves feedback from the third meeting. It also focuses on planning for the upcoming family meeting (meeting 5). The fifth meeting is the Family Talk in which all family members participate and are encouraged to talk with each other about their own experiences, culminating in the development of a plan for continuing to support and maintain family communication. The sixth meeting is a follow-up with parents, and sometimes children, focusing on how the intervention was experienced and how the family can move forward regarding communication and parenting.

### Settings and participants

Families were recruited from two specialized palliative home care units in Stockholm, Sweden, during a one-year period (from March 2017 through February 2018). The units offered 24-h services from multi-professional teams. Inclusion criteria were: at least one parent had a life-threatening illness and was enrolled in specialized palliative home care, there was at least one child in the family aged 6–19 years (but all children were invited to participate) and the family members could understand, write and speak Swedish. All families that met the inclusion criteria were identified by a social worker at each unit. In total, 30 families met the criteria and were asked to participate in the study. They were contacted by phone by the interventionists to set up a meeting where the families received information about the study. In total, 20 families with 34 children (response rate: 67%) agreed to participate in the intervention.

### Data collection

Mixed methods (questionnaires and interviews) were used in this study [24]. After FTI, a member of the research group contacted the families by phone to set up a meeting for an interview; the questionnaires were mailed out before the interview or handed over at the interview. In this study, children’s data were used.

#### Study-specific questionnaires

Study-specific questionnaires were developed according to the method of Charlton [25], see also [23]. The questionnaires were aged-adapted and five versions were constructed (children 6–7 years; children 8–12 years; adolescents 13–19 years; healthy parent/other significant adult; ill parent) and all consisted of five parts: sociodemographics, family communication (primary outcome), illness-related information (secondary outcome) and psychosocial wellbeing (secondary outcome). In addition, inspired by Pihkala and co-workers [16], questions about the experiences of participating in FTI were also developed and included.

This study is based on responses to four questions from the last part of the questionnaire: “Talking to Mum/Dad/my sibling(s) was easier/unchanged/more difficult” (three questions) and “My knowledge about my parent’s illness was better/unchanged/worse”.

A total of 23 children answered the questionnaires.

#### Interviews

All children, together with their families, were invited to participate in an interview after the intervention was completed. The researchers were flexible to the children’s and their families’ wishes regarding where the interview was held and whether they wanted to be interviewed together as a family, alone, or in other constellations. All interviews were held in the children’s homes with one or two researchers. The interviews focused on illness-related information, family communication, psychosocial wellbeing and experiences of the structure and content of FTI. A total of 22 children participated in an interview. Twelve children were interviewed together with one or more family member (e.g. the whole family, one or more parent or siblings) and 10 children did the interview on their own. The time span between completion of FTI and the interviews ranged from 5 days to 14 weeks (mean 4 weeks) owing to, e.g., the ill parent’s treatment or death. The interviews with children alone lasted between 6 and 21 min, and the interviews with children and one or more family member lasted between 17 and 81 min; all interviews were tape-recorded and transcribed verbatim.

### Data analysis

#### Questionnaire data

The four study-specific questions were analyzed with descriptive statistics (frequencies), using IBM SPSS Statistics (version 24).

#### Interview data

A conventional content analysis was used to analyze the interviews [26]. As a first step, all authors read the transcripts to get a sense of the whole. As a second step, meaning units were selected and, based on similarities and differences, sorted into codes (first and fourth author). Then, based on similarities in content, the codes were grouped into categories (first, fourth and last author). Lastly, we related the categories to the key concepts of FTI that were of relevance for the children (i.e., “planning how to talk to the children” was excluded) in order to better understand in which areas FTI has shown potential effects for the children (first and last author) (Table 1). All steps were discussed by all authors until consensus was reached.

## Results

### Children’s characteristics

A total of 25 children, aged 8–17 years, from 16 families, participated in this study; 16 were boys and nine were girls. Ten children lived in families categorized as nuclear families, thirteen lived in stepfamilies and two had a single parent. All but four children had siblings. A majority of the ill parents had a cancer diagnosis, and eight parents died during the time of the study (two of them during FTI).

In the text below, the term *younger children* is used for children aged 8–12 years (mean age 10 years), and the term *teenagers* for children aged 13–19 years (mean age 14 years). When the term *children* is used, this refers to all participating children regardless of age.

### Potential effects of FTI

All the categories that were derived from the interviews could be related to the key concepts of FTI: *Bringing knowledge about illness*; *Addressing the needs of the children*; *Breaking the silence together as a family*; and *Continuing the family dialogue, moving on and facing the future.* The only exception was the key concept *Sharing a history together*, which did not fit with any of the data (Table 2). As the results from the questionnaire data were congruent with two of the categories from the interview data, the questionnaire results are interwoven with the text below under the categories “Increased knowledge about illness” and “Improved or unchanged family communication”.

**Table 1:** Examples of quotations, codes, categories and key concepts based on interview data

	Quotations	Codes	Categories	Key concepts
1	It was a bit scary at first ‘cause it felt like... (silence) I got scared and didn’t know much about his (the father’s) illness then... I didn’t know if it was really bad cancer or... more okay cancer... so then I got scared... It was good talking to her (the interventionist) and, and lots of information... yeah, more about dad’s illness and if, y’know, if they could cure it and... and that (8–12 years old)	Increased knowledge about the illness	Increased knowledge about the illness	Bringing knowledge about illness
2	Or yeah, I thought the... arrangements we figured out were really good, especially everything about... school and all, that was such a relief and has helped me (13–19 years old)	School	Getting help with support outside the family	Addressing the needs of the children
3	They do things I can’t cope with, they’ve dealt with them instead of me, like getting hold of a psychologist and so on, showed me who I can turn to and stuff like that (13–19 years old)	Professional conversational therapy		
4	Because... like... you can talk about your thoughts and... and not just, like... ‘cause otherwise you might just walk around thinking about it a lot... and... you could just... you can just... like... I don’t know how to explain it but... (silence). I mean so you don’t just ignore it but you face it... yeah... so you aren’t just in your own little bubble if I can call it that (8–12 years old)	A possibility to put words onto thoughts and feelings	Getting help to find strengths to maintain everyday life	
5	So yeah, this is what I do about most things... things that upset me... yeah, I just try to focus on what’s good (13–19 years old)	Helped to identify strengths		
6	Yeah, we talked about how it’s important to still y’know... yeah but they’ve sort of given me ideas... about how you should... keep moving forward and, like, still do things and so on... yeah... (13–19 years old)	The importance of continuing everyday life outside of the family		
7	I guess it was so you would... so everybody would start talking to each other again... yeah, ‘cause we didn’t talk to each other a whole lot (8–12 years old)	Opening up for conversation among family members	Improved or unchanged family communication	Breaking the silence together as a family
8	Then we were sitting in the kitchen talking about... um... when dad... um... that time when he started coughing blood... yeah, and so it was happening while we were talking about it and then we talked about, like, what we should do if it happened again... yeah, and then we should... first of all call an ambulance (8–12 years old)	Situation related to illness	Preparedness	Continuing the family dialogue, moving on and facing the future
9	Um... what we were talking about... um... um... what she would like to be wearing when she was buried... and, yeah, right, but... like what music and what food we should eat at the funeral and... how we should be dressed and so on... so it was like mostly that stuff, um... yeah, but... (sighs) I mean... it felt good... like we had everything under control... or not everything, but... most things... (8–12 years old) That was I guess we went through your will and a bit about your funeral and how you want that to be (13–19 years old)	Funeral and will		
10	Um... ‘cause if you start talking late (the interviewer has asked if FTI came at the right time) then maybe you’re sort of a bit more prepared, you might say... for what’s going to happen... (8–12 years old)	Death		
11	But me, I notice we’ve started cleaning up more and so on... we already knew how but it wasn’t something we had to do... back then a few years ago, but now we have to... now, when it’s messy we have to clean up (8–12 years old) And we talked about Dad’s sickness and things we divided up that we had to do at our house so it wouldn’t be too much stuff for Dad that he usually did or if he got mad if he had to do things he didn’t want to do so we made a schedule — Yeah really! (8–12 years old)	Helping more with household chores	Continuing everyday life with the family	
12	That we got to talk to each other... and maybe... yeah, not just talking but being together (8–12 years old)	Starting to spend more time together		
13	But then they gave us tips about... that we should go out for walks and talk (13–19 years old)	How to integrate communication in everyday life		

### FTI key concept: bringing knowledge about illness

#### Increased knowledge about the illness

In the questionnaire, after participating in FTI, two thirds (8/12) of the younger children and around half of the teenagers (5/11) reported increased knowledge about their parent’s illness. The other children, regardless of age, reported that their knowledge about the parent’s illness was unchanged, but not lessened (Table 3).

**Table 2:** Categories sorted into the key concepts of FTI

Categories	FTI key concepts
–	Sharing a history together
Increased knowledge about illness	Bringing knowledge about the illness
Getting help with support outside the family	Addressing the needs of the children
Getting help to find strengths to maintain everyday life	
Improved or unchanged family communication	Breaking the silence together as a family
Preparedness	Continuing the family dialogue, moving on and facing the future
Continuing everyday life with the family

**Table 3:** Children’s report on family communication and illness-related information

	Children, 8–12 years, *N* = 12	Teenagers, 13–19 years, *N* = 11
**Support programmes like this one can feel good or bad. How did you feel about it afterward?**		
**Talking to Mum was …**	Easier: 8/12	Easier: 4/11
	Unchanged: 4/12	Unchanged: 7/11
	More difficult: 0/12	More difficult: 0/11
	I don’t have a Mum: 0/12	I don’t have a Mum: 0/11
**Talking to Dad was …**	Easier: 6/12	Easier: 2/11
	Unchanged: 6/12	Unchanged: 7/11
	More difficult: 0/12	More difficult: 0/11
	I don’t have a Dad: 0/12	I don’t have a Dad: 1/11
		Missing: 1/11
**Talking with my sibling(s) was …**	Easier: 4/12	Easier: 2/11
	Unchanged: 6/12	Unchanged: 8/11
	More difficult: 0/12	More difficult: 0/11
	I don’t have a sibling: 1/12	I don’t have a sibling: 1/11
	Missing: 1/12	
**My knowledge about my parent’s illness was …**	Better: 8/12	Better: 5/11
	Unchanged: 4/12	Unchanged: 6/11
	Worse: 0/12	Worse: 0/11

During the interviews, the children described that through FTI they gained new information and therefore increased knowledge about the parent’s illness. Often at the child’s request, information was provided by the interventionists, the parents, and physicians to help the child learn more about their parent’s illness. One younger child described it: *It was good. (*Interviewer*: Why?) Because I learned a lot. (*Interviewer: *What did you learn?) I don’t know... if I think about it I remember... no, but it was much easier about X’s* (the stepmother’s) *illness, or illness* (whispers) *I don’t know what it’s called... so anyway it was easier to understand her illness...* (8–12 years old). Some children explained that learning more about the illness reduced their worries: *It was a bit scary at first ‘cause it felt like...* (silence) *I got scared and didn’t know much about his* (the father’s) *illness then... I didn’t know if it was really bad cancer or... more okay cancer... so then I got scared... It was good talking to her* (the interventionist) *and, and lots of information... yeah, more about Dad’s illness and if, y’know, if they could cure it and... and that...* (8–12 years old)*.* For one younger child, the information seemed not to have been very helpful due to lack of age adaptation: *Yeah, a doctor came to my house and told us... and when she told us we could say things and then she said what it was and so on. (*Interviewer: *Do you remember what the doctor said?) Um... not exactly, there were so many words* (8–12 years old).

### FTI key concept: addressing the needs of the children

#### Getting help with support outside the family

During the interviews, the children’s comments revealed that their participation in FTI helped them to get support from outside their family. The interventionists were described as quick, practical, kind and helpful when it came to supporting the children in their situation at school. For example, they facilitated communication with teachers about things that were important to the children, such as being able to have their phone on during lessons: *Like when they said it was okay for me to have my phone on in class because... not that I use it, but just like I have it with me... um... in case anybody called me and all that... and she arranged that* (13–19 years old)*.* The interventionists also helped the children with professional counselling, guiding them to the right person and sometimes even making the first contact to introduce the child. This was appreciated by the children, who might not have been able to manage it themselves, or know how to go about it: *They do things I can’t cope with, they’ve dealt with them instead of me, like getting hold of a psychologist and so on, showed me who I can turn to and stuff like that* (13–19 years old)*.*

#### Getting help to find strengths to maintain everyday life

FTI helped the children to find strengths to maintain everyday life despite the parent’s illness. Two children described how it was helpful and strengthening to put their thoughts and feelings into words: *Because... like... you can talk about your thoughts and... and not just, like... ‘cause otherwise you might just walk around thinking about it a lot... and... you could just... you can just... like... I don’t know how to explain it but...* (silence)*. I mean so you don’t just ignore it but you face it... yeah... so you aren’t just in your own little bubble if I can call it that* (8–12 years old). Two teenagers also mentioned how FTI helped them identify their own resources, such as thinking positively in difficult times in life: *So yeah, this is what I do about most things... things that upset me... yeah, I just try to focus on what’s good* (13–19 years old). During the interviews, two other children talked about how the interventionists helped them to maintain awareness of activities outside their family and to gain strength from those: *Yeah, we talked about how it’s important to still, y’know... yeah, but they’ve sort of given me ideas... about how you should... keep moving forward and, like, still do things and so on... yeah...* (13–19 years old).

### FTI key concept: breaking the silence together as a family

#### Improved or unchanged family communication

After the intervention, two thirds of the younger children (8/12) reported in the questionnaire that it was easier to talk to their mother, and half (6/12) also stated that it was easier talking to their father. The remainder of the younger children reported that communication with their parents was unchanged. When asked about communication between siblings, half of the younger children, reported that it was unchanged, one third said it was easier (4/11, 1 missing), and one child had no sibling (Table 2). Most of the teenagers reported that family communication was unchanged after FTI, both with their mothers (7/11), fathers (7/11) and siblings (8/11) (Table 2). The remainder of teenagers reported that communication was improved, and no one, regardless of age group, reported that communication within their family was becoming more difficult after participating in FTI.

During the interviews children said that participating in FTI had helped the family members to start talking to each other: *I guess it was so you would... so everybody would start talking to each other again... yeah, ‘cause we didn’t talk to each other a whole lot* (8-12 years old). Children also reported that FTI was helpful because it opened up communication within the family: *Nah, you just get... I mean it turns out okay... gets better, because you get... you’re get more open* (13-19 years old). Some children stated that they were talking more within the family after participating in FTI: Young sibling: *I think we talk a bit more.* Adult sibling: *Exactly.* Young sibling: *Yeah.* Adult sibling: *I think so too.* (Young sibling: 8-12 years old; Adult sibling). FTI also helped the children become more receptive, better at understanding other family members’ non-verbal communication, such as feelings and reactions, and also provided them with new insights: *That we understand each other a bit more, like say why somebody is stressed out or why somebody is... we get more insight into each other’s lives, you might say* (13–19 years old).

### FTI key concept: continuing the family dialogue, moving on and facing the future

#### Preparedness

Throughout the interviews, some children said that FTI had helped them face the future by increasing their preparedness regarding the ill parent, such as planning for the funeral: *What we were talking about... um... um... what she would like to be wearing when she was buried... and, yeah, right, but... like what music and what food we should eat at the funeral and... how we should be dressed and so on... so it was like mostly that stuff, um... yeah, but...* (sighs) *I mean... it felt good... like we had everything under control... or not everything, but... most things...* (8–12 years old). They also prepared by discussing what to do in an emergency situation, reading through the dying parent’s will together, and talking about the imminent death.

#### Continuing everyday life with the family

Another focus in FTI was on how the family could continue everyday life together moving forward. Many children described how FTI had given them practical ideas about what they could do to maintain communication within the family and how to integrate communication into everyday life: Younger sibling: *That was when she said we could talk every Friday.* Older sibling: *Yeah, we’re supposed to have our meetings every Friday.* Younger sibling: *Yeah, and we’re supposed to do something together.* (Interviewer: *What were you going to do on Fridays?*) Younger sibling: *Talk.* Older sibling: *We were supposed to talk about, like, stuff, so everybody would know how everybody else felt, if they were tired or stressed out or...* Younger sibling: *And then every Friday we would also decide what we* (the siblings) *were going to do together* (Younger sibling 8–12 years old; older sibling 13–19 years old). But this pre-planning was not appreciated by everyone, and one teenager expressed concerns that the advice was best suited for the parents: *Like, I don’t want it to be so, um, organized... I want it to be, y’know... still the same feeling as usual... you’re sitting there on a Friday and it’s supposed to be all cosy and nice music and fizzy drinks and all that, and I don’t want to ruin the mood by talking about such serious things... sort of predetermined...* (Interviewer: *Would it be better on another day?*) *Maybe... on Tuesdays... on Fridays I might anyway be out with my friends a lot of the time... then... maybe I can’t be there every week... it could be good to switch days maybe* (13–19 years old). On the other hand, one teenager said it was important to keep communicating within the family since it gave him a feeling of control and a greater understanding of the parent’s illness situation: *It’s easier when you know more about it, when you’re always kept up-to-date with what’s happening, it’s easier to handle ... I mean... oh, help... it’s enough if your parents just keep you posted and say what’s going on... to get a more... it feels better if you know what’s going to happen than if you don’t know what’s happening and you’re just worried* (13–19 years old). The children reported helping out more around the house now, both to make things easier for everyone in the family and to prevent conflicts. They said that FTI had helped initiate these improvements: *And we talked about Dad’s sickness and things we divided up that we had to do at our house so it wouldn’t be too much stuff for Dad that he usually did or if he got mad if he had to do things he didn’t want to do, so we made a schedule — Yeah really!* (8–12 years old). Participating in FTI also led to the children spending more quality time with their siblings and parents, time not related to the illness or household chores. When a 10-year-old was asked what had been the best thing about participating in FTI the child said: *That we got to talk to each other... and maybe... yeah, not just talking but being together.*

## Discussion

To our knowledge, this is the first study reporting the potential effects of FTI with minor children who have a parent with a life-threatening illness, one being cared for in specialized palliative home care. After participating in FTI, the children reported increased knowledge about their parent’s illness and the younger children felt that it became easier to talk to their parents. However, most of the children reported no change in their communication with siblings. Through their participation in FTI, children received help from the interventionists with situations at school and to establish professional counselling. Furthermore, FTI facilitated the beginning of communication between children and their families, helped them to prepare for future illness-related situations, other future events, and to maintain everyday life and minimize conflicts. All of this was appreciated by the children and described as strengthening and helpful.

The interviews revealed that learning more about the parent’s illness gave children better control over and a greater understanding of the situation, which they described as a relief. These results are in line with previous studies, where children from other care contexts have described similar experiences after participating in FTI [15, 17, 18, 22, 27]. Even though one main goal of FTI is to increase the transfer of illness-related information and provide specific knowledge about the illness, not all of the children reported these outcomes. The present study showed that the information given was not always appropriately age-adapted. Bugge et al. [22] and Forrest et al. [28] stress the importance of informing children appropriately and then checking to see that they have understood correctly, as misunderstandings can increase fear and anxiety at the time, and even lead to psychological distress later in life [3].

This study found that FTI helped the children to put their thoughts and feelings into words, think positively in difficult times, and gather strength from life outside of the family. Previous research on resilience has stressed the importance of having friends, a life outside the family, knowledge about the illness, communication within the family, and self-understanding [29, 30]. In this study, FTI increased participants’ awareness of these factors, which can be interpreted as supporting the family to identify strengths and resources, such as building and facilitating resilience, a main goal of FTI [20]. The findings of this study also showed improved communication with the parents, mainly for the younger children, which they described as helpful. This is also in line with one of the goals with FTI [20] and the results of previous FTI studies, which have shown good effects in opening up family communication [15, 22, 27]. However, the present study showed no change in communication between siblings after participating in FTI. Although the intervention is neither intended nor structured to promote communication between siblings (but rather between parents and children or between the parents only, in meeting 1–2, 4), improved sibling communication would have been desirable since children’s relation to each other is also important for the family as a system [8]. However, siblings’ relations to each other have not been studied as much as the adult–child relationship.

No category in this study could be related to the key concept *“sharing a history together”*. One reason for that might be that this is described as a gradually developing understanding, which evolves over time, and the main source of information related to that key concept focuses on the first two sessions where only the parents participate [20]. In addition, the interviews in this study were performed fairly soon after FTI was completed, and the children may not yet have had time to be able to see or reflect on what the family members different stories could mean for them. On the other hand, the structure of FTI gives parents more space to share their history and it might therefore be unrealistic to expect that the children will experience that they are sharing a family history after FTI. The only formal opportunity for children to share their history with the rest of the family is during the family meeting (meeting 5). However, the family meeting mainly focused on other things, such as the parent’s impending death (Eklund R, Kreicbergs U, Alvariza A, Lövgren M: Childrens views are not taken into account in accordance with article 12 of the United Nations convention on the rights of the child in the family talk intervention when a parent is cared for in palliative care. under review). Another potential reason that the children did not report that they “shared their history” in this study might be that during FTI, it is not the children, but the parents, who describe to the interventionists what they believe the children’s situation is like, and what the children need (meeting 1–2). It might therefore be difficult for the interventionists to leave the parents’ perspective of the child behind, to really listen to the children’s own voices and to assume their perspectives during the meeting with the child (meeting 3) and to maintain that perspective at the subsequent family meeting (meeting 5). In other words, FTI primarily involves a child perspective, which means an adult’s external perspective on the children’s experiences and actions, with focus on what adults’ think is best for the child, rather than the child’s own perspective, where the children’s voices are really being listened to (Eklund R, Kreicbergs U, Alvariza A, Lövgren M: Childrens views are not taken into account in accordance with article 12 of the United Nations convention on the rights of the child in the family talk intervention when a parent is cared for in palliative care. under review, [31]).

Children in the present study felt they became better prepared in several contexts after participation in FTI, e.g. the funeral. A previous study with 622 teenagers who lost a parent, shows that 98% wanted to be told when the parent’s death was imminent [32]. Other studies [2, 3], show that children who believed until three days before the death of a parent that the illness was curable, who distrusted the care given to the dying parent, or whose families lacked cohesion, were more likely to suffer long-term psychological distress, years after the loss. Beardslee [20] stresses that by increasing children’s knowledge, breaking the silence and starting communication within the family, and giving children possibilities for moving on and facing the future, the children could be freed from guilt and fear. This may lead to the children remaining strong and doing well in the long run, despite having lived through difficult life situations.

To our knowledge, this pilot study is the first FTI conducted in a palliative care setting that employs mixed methods, which are preferable when evaluating complex interventions [33]. Nonetheless, it has weaknesses. For example, it involves only Swedish-speaking families and has no comparison group. On the other hand, the children’s own assessments were used rather than parental proxy, as the intention was to capture the children’s own experiences. This study shows that FTI was of value to the participating children, and thus may indicate that FTI could be useful to other children. But before these results can be generalized, FTI should be further evaluated in a larger study involving families with minor children and with a family member in palliative care.

## Conclusion

Children living with a parent who had a life-threatening illness and who participated in FTI reported the intervention as strengthening and helpful, with potential effects in terms of better information related to the illness and better family communication. They also reported that FTI facilitated their preparation for the future, decreased family conflicts, and started to build resilience. The results of this study show that FTI is promising for these children. However, FTI would benefit from including children’s perspective to a greater extent in order to achieve all of the goals of FTI. Moreover, the relationship between siblings needs to be given more attention.

## Data Availability

The data used and analyzed in this study are available from the corresponding author on reasonable request.

## Abbreviation

FTI: The Family Talk Intervention
